# Pains and Gains from China's Experiences with Emerging Epidemics: From SARS to H7N9

**DOI:** 10.1155/2016/5717108

**Published:** 2016-07-20

**Authors:** Pengfei Wei, Zelang Cai, Jinwen Hua, Weijia Yu, Jiajie Chen, Kang Kang, Congling Qiu, Lanlan Ye, Jiayun Hu, Kunmei Ji

**Affiliations:** Department of Biochemistry and Molecular Biology, Health Science Center of Shenzhen University, Shenzhen 518060, China

## Abstract

Over the recent decades, China experienced several emerging virus outbreaks including those caused by the severe acute respiratory syndrome- (SARS-) coronavirus (Cov), H5N1 virus, and H7N9 virus. The SARS tragedy revealed faults in China's infectious disease prevention system, propelling the Chinese government to enact reforms that enabled better combating of the subsequent H1N1 and H7N9 avian flu epidemics. The system is buttressed by three fundamental, mutually reinforcing components: (1) enduring government administration reforms, including legislation establishing a unified public health emergency management system; (2) prioritized funding for biotechnology and biomedicine industrialization, especially in the areas of pathogen identification, drug production, and the development of vaccines and diagnostics; and (3) increasing investment for public health and establishment of a rapid-response infectious diseases prevention and control system. China is now using its hard-gained experience to support the fight against Ebola in Africa and the Middle East Respiratory Syndrome in its own country.

## 1. Introduction

The arms race between human immunity and ever-evolving viral pathogens is ancient and abiding. Notable viral pandemics include the small pox (Variola virus) pandemics of Eurasia and North America, the 1918 Spanish flu pandemic (H1N1 virus, ~50 million deaths), and the 1957 Asian flu pandemic (H2N2 virus, ~69,800 deaths). Although humanity has won some battles, such as those with the eradication of small pox, we will never be totally free of the challenges of emerging viruses. Globalization has heightened the risk of rapid widespread viral transmission, especially of viruses transmitted via the respiratory route or close contact.

In the past decade, China experienced four emerging respiratory virus epidemics, namely, those caused by the severe acute respiratory syndrome- (SARS-) coronavirus (Cov) and the H5N1, H1N1, and H7N9 viruses (Table 1). The SARS-Cov originated in southern China in 2002 [1]. It is believed that the virus was transferred to humans from an animal vector (e.g.,* Paguma larvata*) sold in Chinese food markets [2]. All told, there were 5,327 cases and 348 deaths attributed to SARS in mainland China from November 2002 to June 2003 [3]. Similarly, it appears that the avian flu virus H5N1 was transmitted to humans from poultry, with the first patient being reported in Hong Kong in 1997 [4]. There have been a total of 47 laboratory confirmed human cases (30 deaths) of H5N1 in mainland China (as of December 4, 2014) [5]. The first outbreak of H7N9, also transferred from poultry, occurred in eastern China in March 2013 [6]. There were 469 laboratory confirmed human cases (182 deaths) of H7N9 infection by the end of 2014 [7]. Meanwhile, the H1N1 virus was derived from several circulating swine viruses in North America and was then imported to China, rather than being of local origin, resulting in 128,033 laboratory confirmed cases (805 deaths) in China, but fortunately with a relatively low mortality rate (0.6%). Additionally, some sporadic cases of influenza caused by the emerging viruses of H10N8 (3 cases/2 deaths) and H5N6 (2 cases/1 death) have been observed in China, raising concerns about potential emerging influenza outbreaks in the future [8, 9].

The SARS tragedy brought great pain to China and the world. However, as the ancient Chinese proverb says, “failure is the mother of success.” Realizing the devastating effects of the emerging epidemic, the Chinese government endeavored assiduously to develop a systematic infectious disease control strategy. Indeed, as stated by Stone, “No country has taken stricter measures than China to protect residents from pandemic swine flu,” and China was the first nation to vaccinate its people against H1N1 virus [10]. During the 2013 H7N9 epidemics, Chinese authorities were commended globally for their rapid response and transparency. Over the last decade, China has accumulated valuable experience in the ongoing war to control emerging virus outbreaks. Therefore, here, we analyze the lessons of the aforementioned outbreaks and the systematic strategy developed by China as a result. We believe that it would benefit human security and public health for the Chinese experiences to be shared internationally, especially as we now face new threats, such as Ebola, Middle East Respiratory Syndrome (MERS), and emergent influenza viruses.

## 2. Lessons from the SARS Tragedy

The SARS virus spread rapidly in China (Table 1) and internationally, causing global panic. By June 11, 2003, 32 countries and regions were affected, with 8,437 cases and 813 deaths worldwide, including 3,110 cases and 465 deaths outside of China.

Early identification and early isolation are imperative for infectious disease control. Several mistakes and shortcomings in China's handling of SARS can be identified. Firstly, there was not a well-functioning nationwide infectious diseases surveillance system, nor were there effective regulations to prevent disease dissemination. The government did not recognize the severity of the SARS epidemic and did not inform the public promptly. Initially, SARS patients and their close contacts were not isolated, enabling the virus to spread quickly. China missed its best opportunity to contain the SARS epidemic in a timely manner.

To make matters worse, the Chinese government did not organize effective collaborations to facilitate identification of the offending pathogen as soon as possible. Autopsies of several early SARS patients revealed a Chlamydia coinfection, leading the medical examiner to conclude rashly that the lethal disease could be attributed to Chlamydia infection, which misled Chinese authorities and delayed recognition of SARS. Another independent research group obtained evidence suggesting that the disease outbreak was due to a novel coronavirus, but their work did not gain the attention of the Chinese government [11]. Ultimately, WHO organized an international collaborative endeavor in which 11 research groups embarked on a hunt for the culprit, and one month later the SARS-Cov was identified by the scientists in America, Germany, Hong Kong, and Canada [12–15]. This experience instructs us that governments should refrain from administrative interference in public health investigations and promote extensive collaborative research—through appropriation of funds and sharing of samples and technology—to facilitate identification of emergent pathogens as soon as possible.

SARS-Cov can be transmitted through direct and indirect contact of the mucous membranes of the nose, mouth, or eyes [16]. In the early stages of the SARS epidemic, health care workers wore masks that protected only the nose and mouth because they did not realize that the virus may be also transmitted through the eyes. Unfortunately, 1,002 Chinese health care workers were infected, representing 18.8% of the total cases [17]. Problem was solved after health care workers began wearing isolation gowns. Additionally, SARS patients were given excessive amounts of steroid hormones in some Chinese hospitals, leading to grave sequelae, including bone necrosis, which has had lasting consequences for many survivors.

Unfortunately, laboratory biosafety regulations were poorly enforced in China in the early 2000s, allowing a renewal of the SARS crisis. In March of 2004, two cases of laboratory-acquired SARS were reported [11]. These infections were transmitted to seven additional people, one of whom died as a result [11]. The SARS tragedy compelled the Chinese government to reform its administrative rules, promote biomedical technology, and enhance its public health systems.

## 3. Sustaining Government Administration Reforms

Promptly after the SARS epidemic, the Chinese government accelerated the establishment of an effectual and national unified management system for public health emergencies and enacted two laws: the Regulation on Public Health Emergency and the Measures for the Administration of Information Reporting on Monitoring Public Health Emergencies and Epidemic Situation of Infectious Diseases [18, 19]. In addition to defining the standards and grades of public health emergencies, these laws support the construction of command systems and clarify the responsibilities and the leadership role of the chief executive of central and local governments previously held by the Centers for Disease Control and Prevention (CDC). Accordingly, the executive capacity of the command systems has been much improved. Importantly, China also established an emergency information dissemination system to enable timely (within 2 hours), accurate, and comprehensive release of information. Moreover, both central and local governments are now expected to be prepared for a public health emergency response (e.g., techniques, personnel, materials, and management preparedness).

In 2004, China revised the Prevention and Treatment of Infectious Diseases Law, adding SARS and avian flu as notifiable diseases and revising the law to comply with the principal rules of infectious disease prevention and control (i.e., infection source control, interruption of route of transmission, and susceptible people protection) [20]. The law adheres to the “five early” principle of early detection, diagnosis, reporting, isolation, and treatment. Early isolation can restrain contagion. The law applied China's experience in emerging epidemics to prevent and control 37 infectious diseases.

The Chinese Ministry of Health (CMH) issued a technical guide for avian flu prevention and control in 2004 [21]. It requires that suspected and confirmed cases be handled quickly at designated hospitals with the equipment to prevent nosocomial infection. The epidemiological and etiological data of patients should be acquired to enable determination of human-to-human transmission capacity. The guide suggests that persons exposed to dead poultry infected by avian flu virus be isolated and observed for 7 days. In order to control zoonotic infectious diseases, China revised its Law on Animal Disease Prevention in 2007, adding an animal epidemic surveillance and reporting system for timely disclosure of animal epidemics and providing compensation to farmers for economic loss due to culling infected or potentially infected poultry [22].

Guided by the aforementioned laws, a series of social innovations enacted after the SARS epidemic have improved China's ability to combat emerging diseases. The CMH issued a swine flu prevention guide on April 29, 2009, 12 days before the first reported H1N1 case [23]. On April 3, 2013, 4 days after the first H7N9 confirmed case, the CMH also issued a nosocomial H7N9-infection prevention guide [24]. Administrative reforms resulted in better handling of H5N1, H1N1, and H7N9 relative to SARS. Importantly, in keeping with its move toward greater transparency, after confirmation of the first H1N1 case on May 11, 2009, China posted patient zero's travel information publicly on the same day [25]. Likewise, after confirming the first H7N9 avian flu case on March 30, 2013, China published detailed information about the patient's medical consultation [26]. Transparency helps to subdue rumors and maintain social stability.

## 4. Scientific and Technological Progress

With the aim of improving prevention and control of viral outbreaks, the Chinese government has been investing continually in the advancement of science and technology since 2003, including the appropriation of more than 12 billion RMB for research and development related to combating SARS, influenza, and other major infectious diseases [27]. Meanwhile, China has built 11 national technology platforms, 11 national research centers, 6 national key laboratories, and 2 national engineering laboratories. In 2010, the Chinese National Influenza Centre was designated as a WHO Collaborating Centre for Reference and Research on Influenza. All these laboratories and funding contributed to application of advanced technologies in preventing and controlling infectious diseases.

Above all, quick identification of pathogens is a prerequisite to controlling emerging epidemics. To achieve it, China has developed state-of-the-art pathogen isolation and identification technologies such as high-throughput sequencing method. In contrast to the SARS-Cov debacle, H7N9, H10N8, and H5N6 were identified within China [28–30]. BGI, a Chinese company, helped Germany sequence the pathogen* Escherichia coli* O104:H4 within a week using high-throughput sequencing technology in 2011 [31]. Meanwhile, Chinese researchers exploring the genesis and source of emerging viruses have found that bats are natural reservoirs of SARS-like coronaviruses and have demonstrated that domestic fowl play an important vector role for H5N1 and H7N9 [4, 32, 33].

The government encourages the development of diagnostic reagents, vaccines, and medicines as well as prophylactic equipment (e.g., infrared thermometers). China's national vaccine regulatory system was confirmed to meet WHO standards in 2011 [34]. China has developed SARS, H5N1, H1N1, and H7N9 vaccines (Table 1) and became the first country to use an H1N1 vaccine [35]. China now produces oseltamivir (like Tamiflu®) and peramivir (like Rapivab®), obviating the need to import antivirals [35].

China's improvements in research funding and technical capabilities have led to a series of important findings. For example, Chinese researchers have revealed the crystal structures of key viral proteins (e.g., SARS-Cov protease, H1N1 neuraminidase N1, and H5N1 polymerase PA_C_-PB1_N_ complex) [36–38], which is useful for drug design, and discovered an oseltamivir-resistance mechanism in H7N9 [39]. A traditional Chinese medicine (TCM) herbal formula was confirmed to reduce H1N1 influenza-associated fever safely and with efficacy similar to that of oseltamivir in a randomized clinical trial [40].

## 5. Increasing Investment and Public Health System Improvements

Chinese public health funding increased by more than 10-fold from 10.6 billion RMB in 2003 to 110.2 billion RMB in 2012 [41]. In 2013, there were 3,490 CDC branches, with 193,000 staff members, and 2,728 laboratories in animal disease prevention and control institutions in China, enabling rapid-response infectious diseases prevention and control [41]. By 2013, China had set up 3,486 stations to monitor 28 kinds of infectious diseases and four common animal vectors (i.e., mosquitoes, flies, cockroaches, and rats) [41]. Infectious disease surveillance of Entry-Exit personnel and commodities is now carried out at 285 customs and 168 international travel healthcare centers. Additionally, China has established 3,088 public health supervision institutions and a nationwide animal infectious diseases surveillance system [41].

By 2013, infectious diseases departments had been established in 2,243 Chinese hospitals. From 2004 to 2012, the numbers of sickbeds and registered infectious diseases physicians increased by 64% and 58%, respectively [41]. In 2007, the national immunization program increased the number of free vaccinations from 6 to 14. In 2014, China's basic medical security system covered >96% of the Chinese population [41]. Additionally, China has built the largest web-based reporting system of infectious diseases and public health emergencies in the world, enabling the reporting of 37 notifiable diseases reporting delay to be reduced from 5 days to within 4 hours [41]. Hence, China's public health system has been enhanced greatly, improving the ability to fight emerging epidemics.

## 6. China Supports Africa in Combating Ebola

Worldwide cooperation is necessary to combat the current Ebola epidemic. As of June 2015, nine countries have been affected by Ebola (27,273 cases/11,173 deaths) [42]. Based on its recent experiences with epidemics, China has provided comprehensive support to West African nations battling Ebola, including provision of capital, anti-Ebola materials, testing and treatment support, tactical rapid-response training, and collaborative research. China placed approximately 1000 medical experts in West Africa, helped build an Ebola diagnosis and treatment center and a biosafety laboratory, and trained more than 13,000 local medical care and community workers [43].

In December 2014, Chinese-made Ebola diagnostic kits were made available [44]. In March 2015, China's first Ebola vaccine (the first to be based on the 2014 Zaire strain instead of the 1976 Zaire strain) passed its first phase I clinical trial and is being produced in a lyophilized form that is more stable than prior aqueous-form vaccines [45]. China is producing a therapeutic chimeric antibody agent, MIL-77, which is amenable to large-scale production via mammalian cell expression. It was reported that a 25-year-old British nurse infected with the Ebola virus was treated successfully with MIL-77 without adverse effects [46].

## 7. Future Perspective in China

On May 26, 2015, a 43-year-old Korean man infected with MERS travelled by air from Seoul to Huizhou in Guangdong Province, China [47]. After being notified by the WHO about this traveler, the local health authority responded immediately by isolating him and providing him with curative treatment. A group of 1364 people who were identified as having been in contact with this patient were quarantined rapidly and no additional cases of MERS infection were reported in the area [47]. The aforementioned strategies and experience helped Chinese government to promptly prevent the viral transmission.

Because of the regional socioeconomic imbalances in China, more healthcare funding is needed in rural areas. It will be important to construct a better epidemic disease surveillance system. Additionally, to limit the risks of transmission of viruses from animal vectors to humans, the government should restrict the consumption of wild animals, centralize poultry slaughtering, secure the cold-chain transportation system, and abolish the sale of live poultry near residential areas because viruses, such as H7N9, can be transmitted in poultry markets or during at-home killing.

Legislation is needed to ensure that research on highly contagious pathogens is tightly regulated and conducted in high-security facilities. Man-made viruses—such as the H5N1-H1N1 hybrid, which exhibits a high transmission capacity and high mortality rate—should be taken seriously. Additionally, owing to its continuous economic growth and biotechnological progress, China should be an active participant in combating emerging epidemics worldwide, such as MERS and Ebola.

In conclusion, the SARS tragedy compelled the Chinese government to reform its administrative rules, promote biomedical technology, and enhance its public health systems. These improvements involve three fundamental, mutually reinforcing components: (1) enduring government administration reforms, including legislation establishing a unified public health emergency management system; (2) prioritized funding for biotechnology and biomedicine industrialization, especially in the areas of pathogen identification, drug production, and the development of vaccines and diagnostics; and (3) increasing investment for public health and establishment of a rapid-response infectious diseases prevention and control system. China's experience in combating emerging epidemics would benefit human security and public health over the world if shared internationally.

## Figures and Tables

**Table 1:** Summary of epidemics caused by emerging respiratory viruses in China from 2003 to 2014^*∗*^.

Characteristic	SARS-Cov	H5N1	H1N1	H7N9
Country of origin	China	China	Mexico	China

1st case in China	Guangdong, November 2002	Hong Kong, May 1997	Sichuan, May 2009	Shanghai, February 2013

Viral genome	Positive-sense, ss RNA	Negative-sense, ss segmented RNA	Negative-sense, ss segmented RNA	Negative-sense, ss segmented RNA

Pathogen identification	WHO declared SARS-Cov as the pathogen, April 16, 2003	National Influenza Centre, Rotterdam, The Netherlands; National Institute for Medical Research, London, UK; CDC, USA, August 1997	CDC, USA, April 15, 2009	CDC, China, March 29, 2013

Epidemiology in China	5,341 cases 349 deaths DR, 6.5%	47 cases 30 deaths DR, 63.8%	128,966 cases 891 deaths DR, 0.6%	349 cases 136 deaths DR, 38.9%

Human-to-human transmission	Yes	Limited	Yes	Limited

Genesis/source	Bats	Domestic poultry	Swines	Domestic poultry

Diagnostics in China	Real-time PCR	ELISA/real-time PCR	ELISA/real-time PCR	Real-time PCR

Drugs in China	No specific medication	Oseltamivir, amantadine, and rimantadine; TCM	Oseltamivir and zanamivir; TCM	Oseltamivir and zanamivir; TCM

Vaccines in China	Inactivated vaccine Phase I clinical trials finished in 2004	Inactivated vaccine approved in 2008 for storage, not for public sale	Inactivated vaccine approved for market in 2009	Inactivated vaccine approved for clinical trial in 2014

^*∗*^Data from WHO, Chinese Food and Drug Administration, and CMH.

ss: single-stranded; DR: death rate; PCR: polymerase chain reaction; and ELISA: enzyme-linked immunosorbent assay.
